# Sponge-liked Silica Nanoporous Particles for Sustaining Release and Long-Term Antibacterial Activity of Natural Essential Oil

**DOI:** 10.3390/molecules28020594

**Published:** 2023-01-06

**Authors:** Huazhang Lai, Shuiyan Chen, Xiaoyu Su, Xiaoying Huang, Qin Zheng, Ming Yang, Baode Shen, Pengfei Yue

**Affiliations:** Key Labolatory of Modern Preparation of TCM, Ministry of Education, Jiangxi University of Chinese Medicine, 1688 Meiling Avenue, Nanchang 330004, China

**Keywords:** nanoporous silica, *Chimonanthus nitens Oliv*. essential oil, sustained release, anti-bacterial effect

## Abstract

To improve the sustained release and long-term antibacterial activity of *Chimonanthus nitens Oliv.* essential oil (CEO), novel sponge-liked nanoporous silica particles (SNP) were synthesized via the soft template method, which was employed as a biocompatible carrier to prepare spong-liked nanoporous silica particles loading with CEO (CEO-SNP) through physical adsorption. The structure and properties of the samples were characterized via N2 adsorption/desorption measurements, thermogravimetry (TGA), Fourier transform infrared, SEM and TEM. The result showed that the SNP exhibited an excellent loading capability of CEO up to 76.3%. The thermal stability and release behavior of the CEO were significantly improved via the physical adsorption of the SNP materials. The release profile of CEO was in accordance with the first-order kinetic model, which meant that the release mechanism was drug Fick’s diffusion. The antibacterial evaluation results demonstrated that the CEO-SNP exhibited strong antibacterial activity against *S. aureus*, *E. coli* and *P. aeruginosa*. The antibacterial results have shown that the CEO-SNP could destroy the cell structure of bacteria, and result in the generation of oxidative stress and the release of nucleic acid. After storage of 30 d at 25 °C, the CEO-SNP still had the stronger antibacterial activity towards *S. aureus*, *E. coli* and *P. aeruginosa* in comparison with CEO. Therefore, the sponge-like silica nanoporous particles seemed to be a promising carrier for long-term stability and antibacterial delivery of CEO.

## 1. Introduction

Given the large challenge of bacteria resistance caused by the chronic use and overuse of conventional antibiotics, there is great need to seek alternative natural antibiotics that are effective for prevention and treatment of bacterial infection [1]. Essential oils are gaining increasing attention as good alternatives owing to their significant antibacterial activity against bacterial infections [2]. Essential oils (EOs), a complex mixture with higher volatility extracted from aromatic plants, have numerous applications in pharmaceutical, food and cosmetic industries [3,4,5,6]. Because of its strong antibacterial, anti-inflammatory and antioxidant effects, EOs have a wide application in the treatment of many diseases such as bacterial infection, rheumatoid arthritis, anxiety and depression [7,8]. *Chimonanthus nitens Oliv.* essential oil (CEO) is mainly composed of several active components such as linalool, α-pinene and eucalypto, is derived from *Chimonanthus nitens Oliv.* and is used as a folk medicine for the treatment of colds and influenza [9,10]. CEO was reported to possess anti-inflammatory, anti-bacterial and antioxidant activities [11], and seems to be a promising natural antimicrobial.

Unfortunately, the development of Eos as natural antibiotics are largely restricted, owing to their special properties such as volatile nature and hydrophobicity [12]. Furthermore, EOs are unstable and easily oxidized when exposed to light, oxygen and high temperature under environmental conditions [13,14]. In order to tackle with the abovementioned limitations of EOs, encapsulation of EOs by different techniques could increase the bioavailability of EOs and improve their chemical stability while reducing their volatility and hydrophobicity. Many formulation strategies have been currently employed for the potential encapsulation of EOs, including cyclodextrins inclusion, microspheres, polymeric nanoparticles and lipid nanocarriers, etc. [15,16,17,18]. However, there were some drawbacks for these formulation approaches, such as special requirements for molecular size or structure of the EOs, unexpected release behavior of EOs caused by the collapse of microspheres and nanoparticle, low drug-loading and/or potential toxicity of encapsulating excipients. Cyclodextrin-based nanosponges [19,20,21] and porous metal-organic frameworks [22,23] also have been used to improve the stability of volatile molecules and prolong their release. However, their loading capacity is relatively low and affected by the degree of crosslinking [24]. Hence, there is a growing need for novel strategies to improve the stability and antibacterial effect of EOs.

In recent years, as an alternative to cyclodextrin -based nanosponges, polymeric materials and porous metal-organic frameworks and nanoporous silica particles (NP) were biocompatible nanoporous materials with the pores size of 2–10 nm, and have been used as inorganic scaffolds for the storage and release of drugs and organic molecules [25,26]. NP provide unique features such as high thermal stability, biocompatibility, high drug-loading and large surface area, a uniform porous structure and adjustable pore sizes [27,28,29]. The specific size and adjustable polarity of NP can be synthesized by varying the silica source to surfactant templates proportion [30,31,32,33,34,35]. Encapsulation of EOs into the NP can improve their stability and water solubility, and provide a longer-term efficacy by controlled release. Janatova and his coworkers encapsulated volatile EOs into nanoporous silica material MCM-41 and provided long-term effects by controlled release and ease of application [36]. Fan and Gao groups prepared an amino-functionalized nanoporous silica loading tea tree oil and exhibited longer-lasting anti-bacterial activity [37,38]. However, until now, there has been no report using nanoporous silica particles to improve the stability, release and antibacterial activity of CEO derived from *Chimonanthus nitens Oliv*.

**Figure 1:**
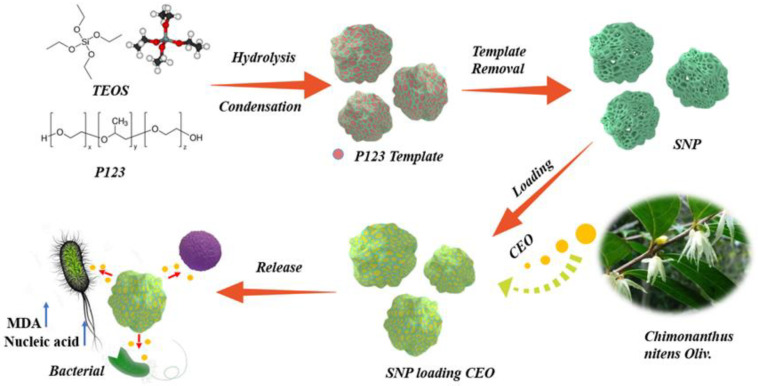
Schematic image of sponge-liked silica nanoporous particles loaded with *Chimonanthus nitens Oliv*. essential oil (CEO-SNP).

In this study, natural CEO was used as model drug. As illustrated in Figure 1, to improve the stability of CEO, novel sponge-liked silica nanoporous particles (SNP) were prepared, and CEO was encapsulated into it through physical adsorption. The effects of SNP on the release performance and stability of CEO were thoroughly investigated, and the antibacterial activity and antibacterial mechanism of SNP towards *Staphylococcus aureus* (*S. aureus*), *Escherichia coli* (*E. coli*) and *Pseudomonas aeruginosa* (*P. aeruginosa*) were systemically investigated in this study.

## 2. Results and Discussion

### 2.1. Characterization of SNP Loading Chimonanthus nitens Oliv. Essential Oil (CEO-SNP) and CEO-SNP

**Figure 2:**
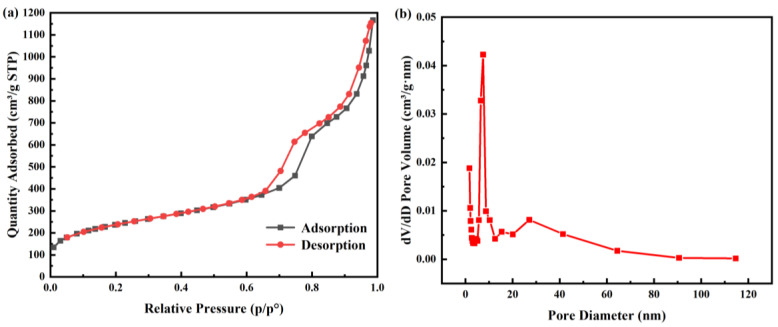
(**a**) N2 adsorption/desorption isotherms of SNP and (**b**) pore size distribution of SNP.

SNP with sponge-liked pore structures was successfully synthesized. The specific surface area, pore size distribution as well as pore volume of SNP and CEO-SNP were detected through Brunauer-Emmet-Teller (BET) analysis. As shown in Table 1, the BET surface, pore volume and pore diameter of SNP were 815.7278 m^2^/g, 1.804470 cm^3^/g and 8.7026 nm, respectively. Compared with other studies on NP-encapsulated essential oil, SNP has a larger pore volume, which can provide more capacity for the loading of essential oil [37,39,40]. As shown in Figure 2a, the N2 adsorption/desorption isotherms of SNP belonged to a typical Langmuir IV isotherm, confirming the existence of a nanoporous structure [41]. Figure 2b shows that the occurrence of capillary condensation in the range of P/P0 = 7.0~10.0 demonstrates that the presence of a mainly small pore size.

**Figure 3:**
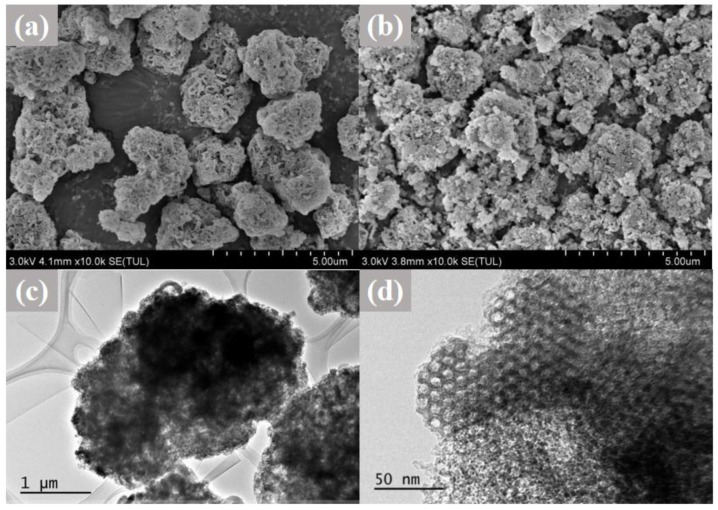
Scanning electron microscope (SEM) images of SNP (**a**) and CEO-SNP (**b**), and transmission electron microscope (TEM) images (**c**,**d**) of SNP.

**Figure 4:**
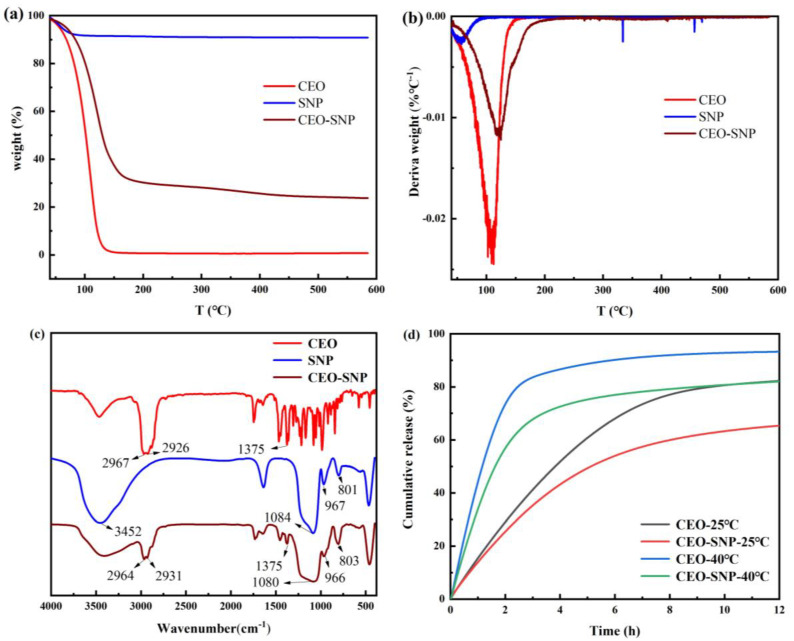
The TGA (**a**) and DTG (**b**) curves and FTIR spectra (**c**) of CEO, SNP and CEO-SNP. Drug release curves (**d**) of CEO and CEO-SNP at 25 and 40 °C.

The morphology of SNP was shown in Figure 3. SNP seemed to be the irregular particles with a clear pore structure. Figure 3a illustrates that SNP possessed a sponge-liked shape with an obvious porous structure on the surface. Compared with SNP, no significant difference in morphology can be observed for CEO-SNP (Figure 3b), indicating no morphology change during the loading process of CEO. TEM showed that SNP possessed an ordered hexagonal pore structure (Figure 3c,d).Both SEM and TEM demonstrated a large number of uniform pores of SNP, which was consistent with the characteristic of the pore structure of nanoporous silica [42]. The abundant pores could contribute to the encapsulation of CEO [37,43], which was evidenced by the TG analysis (Figure 4a).

Figure 4a shows the TG analysis results of CEO, SNP and CEO-SNP. About 9.2% weight loss was observed from 40 °C to 110 °C for SNP, which could be due to the evaporation of bound water molecules. No weight loss was found in the subsequent temperature range indicates that the good thermal stability of SNP and the P123 template has been successfully removed from SNP. An obvious weight loss from 40 °C to 160 °C could be observed, explaining the volatilization of CEO, and the residual content of CEO at 160 °C was only 0.9%. As shown in the TGA curve corresponding to CEO-SNP, a large weight loss event appeared in a temperature range from 40 °C to 210 °C, which was mainly attributed to the loss of CEO in the SNP channel. Furthermore, the weight loss was 76.3%, which corresponded to the loading content (LC) of CEO in CEO-SNP, indicating that the LC of CEO-SNP was 763 mg/g. The LC of CEO-SNP was remarkedly higher than those of other related studies [40,44], and this excellent loading performance for essential oils could be explained by the sponge-like structure of SNP.

The DTG results of CEO, SNP and CEO-SNP were shown in Figure 4b. It could be observed that the decomposition peak temperature of CEO was from 108 °C to 160 °C. Compared to CEO, CEO-SNP decomposed from 124 °C to 210 °C. These demonstrated that the adsorption of CEO by SNP could significantly the improve stability of CEO, which is dependent on the Vander force and/or hydrogen bonding effect.

FTIR spectroscopy was used to determine the chemical structures of CEO, SNP and CEO-SNP. As displayed in Figure 4c, the bands located at 2967 cm^−1^ and 1375 cm^−1^ in the CEO spectra could be respectively assigned to the C–H stretching and bending vibrations of methyl. For SNP spectra, the absorption bands of Si–O–Si appeared at 1084 cm^−1^ and 801 cm^−1^, and the bands corresponding to the stretching and bending vibrations of Si–OH were found at 3452 cm^−1^ and 967 cm^−1^. CEO-SNP exhibited the corresponding bands of SNP at 1080 cm^−1^, 966 cm^−1^ and 803 cm^−1^ and the bands of CEO at 2964 cm^−1^ and 1375 cm^−1^, indicating that CEO was successfully loaded into the SNP carrier.

### 2.2. Sustained Release Evaluation of CEO-SNP

Figure 4d displays the release curves of CEO and CEO-SNP. It could be seen that the cumulative release of CEO at 25 °C and 40 °C reached 50% at 3.82 h and 1.16 h, respectively. In addition, the cumulative release reached 82.25% (25 °C) and 93.28% (40 °C) at 12 h, respectively. However, the release curve of CEO-SNP revealed that the release time to reach 50% at 25 °C and 40 °C was 5.12 h and 1.63 h, respectively. Similarly, at the above two temperatures, the cumulative s for 12 h were 65.39% (25 °C) and 82.02% (40 °C), respectively. Raising the temperature led to a substantial increase of the release rate of CEO, resulting from the fact that the CEO volatility increased with the rising temperature. Furthermore, these results demonstrated that CEO-SNP exhibited the relatively sustained release profile in comparison with CEO. Therefore, the volatilization rate of CEO could be effectively reduced by SNP encapsulation, which could be attributed to the nanopores of SNP and hydrogen bonding between the silanol groups and CEO that slows the release of CEO.

**Figure 5:**
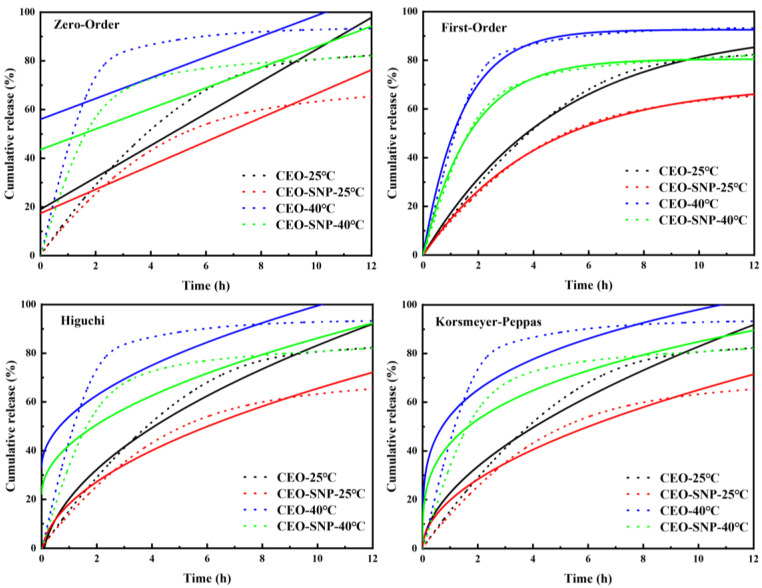
Model fitting curves of CEO, CEO-SNP at 25 and 40 °C. The dashed line represented the original experimental data and the straight line represented the fitting curve.

To further explain the release mechanism of CEO, the release result was fitted by using four different kinetic models (Table 2). The fitting curve is shown in Figure 5 and Table 2. The results showed that the model with the highest value of R^2^ was generally recognized as the best fitted model for the release profiles [45]. Table 2 shows that the values of R^2^ for First-order kinetic model (R^2^ > 0.99) was higher than other models, indicating it was the most suitable model for the sustained release behavior of CEO and CEO-SNP. This meant that the release of CEO and CEO-SNP was driven by the concentration gradient [46].

### 2.3. Stability Evaluation of CEO-SNP

**Figure 6:**
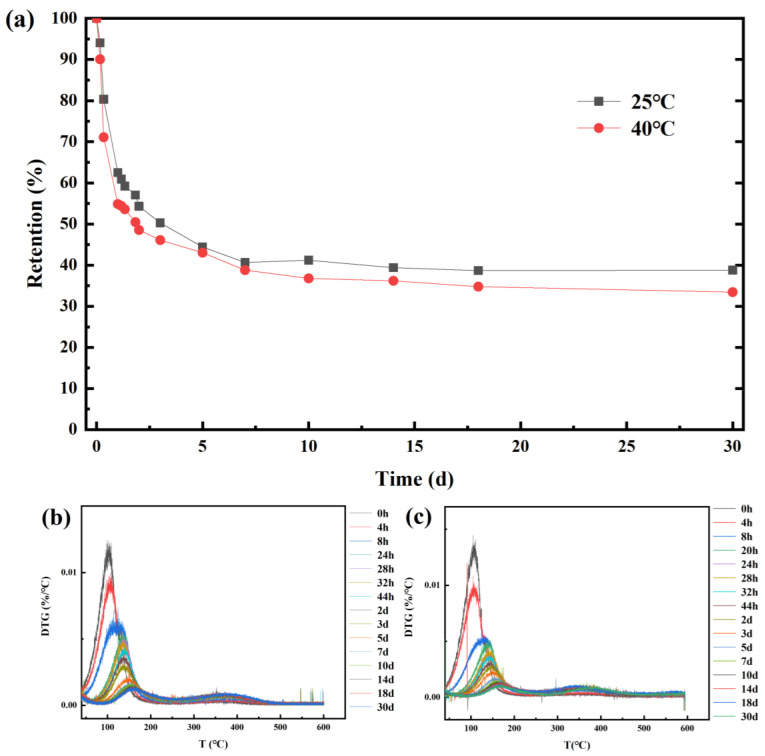
The curve of retention (**a**) of CEO-SNP at different time in environment at 25 °C and 40 °C. The curve of DTG of CEO-SNP at different time in environment at 25 °C (**b**) and 40 °C (**c**).

As shown in Figure 6a, the stability of CEO-SNP was evaluated at 25 and 40 °C. The results of CEO-SNP implied that the volatility of CEO from CEO-SNP could be divided into three distinct phases. At the first phase (0–24 h), the retention at 25 °C and 40 °C decreased rapidly to 62.52% and 54.87%, respectively, which might be attributed to the CEO volatilization from the surface of SNP. Subsequently, the volatilization rate of CEO from the CEO-SNP was relatively slow during the period from 24 h to 7 d, and the retention of CEO-SNP decreased from 62.52% to 40.66% at 25 °C, and from 54.87% to 38.78% at 40 °C. These could be attributed to the volatilization of CEO adsorbed on the shallow pores of the SNP. Ultimately, the volatilization of CEO reached an appreciably slow-release phase at 25 and 40 °C. It could be the reason that CEO was required to overcome greater resistance in order to volatilize from the deep pore of SNP. For Figure 6b,c, the maximum weight loss temperature of CEO-SNP increased significantly in comparison with those of CEO, indicating that the stability of the remaining essential oil was gradually improved, which was in accordance with the release results of CEO-SNP.

### 2.4. Antibacterial Performance Analysis

#### 2.4.1. Determination of Minimum Bactericidal Concentration (MBC)

**Figure 7:**
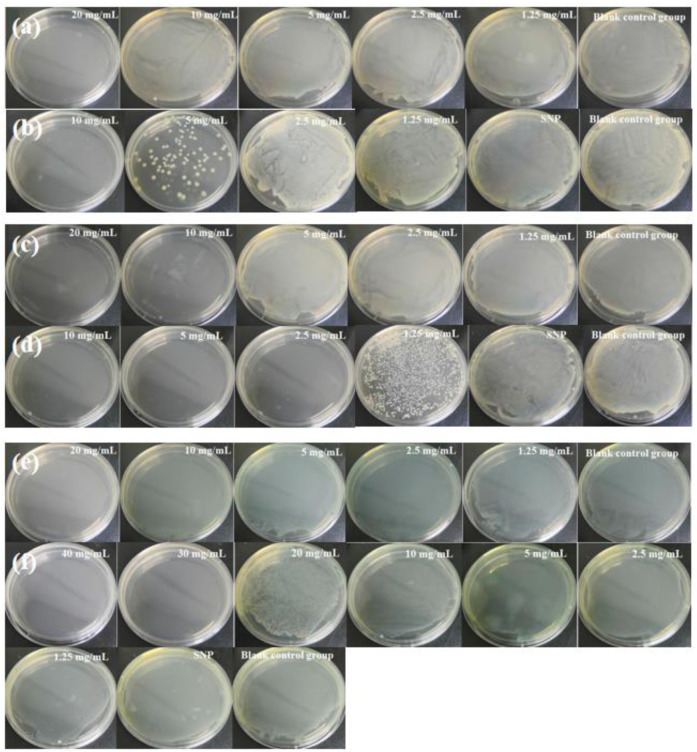
Image of the minimum bactericidal concentration test results of CEO and CEO-SNP against *E. coli* (**a**,**b**), *S. aureus* (**c**,**d**) and *P. aeruginosa* (**e**,**f**), respectively. (**a**,**c**,**e**) represents the antibacterial effect of CEO, whereas (**b**,**d**,**f**) represents the antibacterial effect of CEO-SNP.

The antimicrobial activities of CEO and CEO-SNP were evaluated by determining their MBC against *S. aureus* (gram-positive), *E. coli* (gram-negative) and *P. aeruginosa* (gram-negative). The results are displayed in Figure 7. CEO had strong antimicrobial properties against Gram-positive *S. aureus* as compared to Gram-negative *E. coli* and *P. aeruginosa*, evidenced by the MBC of CEO for *S. aureus* (10 mg/mL), *E. coli* (20 mg/mL) and *P. aeruginosa* (20 mg/mL). The results were consistent with the previous reports that generally, Gram-negative bacterium tended to be less susceptible to EOs than Gram-positive ones [47,48]. The reason might be that Gram-negative organisms possessed more complex and rigid outer membrane with rich lipopolysaccharide (LPS), strongly restricting diffusion of hydrophobic compounds such as EOs through it [49,50]. In contrast, the density of the peptidoglycan wall surrounding Gram-positive bacteria was insufficient to prevent drug molecules from entering the cellular membrane [51]. As shown in Figure 7b,d, the MBC of CEO-SNP towards *E. coil* and *S. aureus* was 10 mg/mL and 2.5 mg/mL, respectively. It could be seen that the MBC of CEO-SNP was lower than that of CEO, implying that encapsulation of CEO into SNP significantly enhanced its bactericidal activity. It could be attributed to the fact that CEO-SNP made easy contact with the bacteria and possessed more contact sites with the cell membrane provided by the larger specific surface area of SNP. The solubility of CEO in water increased, owing to the encapsulation of SNP, which could facilitate CEO to penetrate the bacterial cell membrane [52]. Furthermore, CEO could be effectively protected from degradation or evaporation, and gradually released to the media [53]. Strikingly, for Figure 7e, the MBC value of CEO-SNP against *P. aeruginosa* was 30 mg/mL, larger than the MBC value of CEO, possibly due to the tolerance of *P. aeruginosa* to EOs. *Pseudomonads* and *P. aeruginosa*, among the Gram-negative bacteria, were thought to be least sensitive to the action of EOs [54,55]. Moreover, CEO-SNP released slower as compared to pure CEO, which might also lead to higher concentrations of CEO-SNP that are required to achieve the concentration-dependent bacteriostatic effect against *P. aeruginosa* through the SNPs increased solubility of CEO and contact sites with bacterial cell membranes.

#### 2.4.2. Long-Term Antibacterial Effect Test

**Figure 8:**
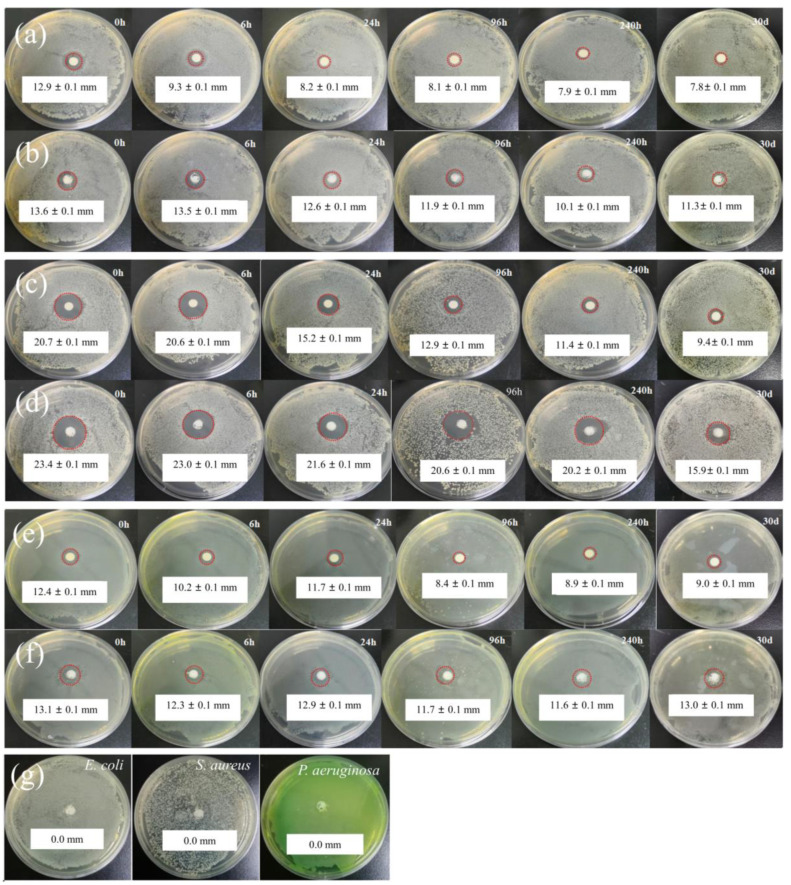
The inhibition zone of CEO (**a**) and CEO-SNP (**b**) on *E. coli* at different release times (0 h, 6 h, 24 h, 96 h, 240 h and 30 d). Inhibition zone of CEO (**c**) and CEO-SNP (**d**) on *S. aureus* at different release times (0 h, 6 h, 24 h, 96 h, 240 h and 30 d). Inhibition zone of CEO (**e**) and CEO-SNP (**f**) on *P. aeruginosa* at different release times (0 h, 6 h, 24 h, 96 h, 240 h and 30 d). Inhibition zone of SNP (**g**) on *E. coli*, *S. aureus* and *P. aeruginosa*.

Figure 8 displays the inhibition zone of CEO and CEO-SNP against *S. aureus*, *E. coli* and *P. aeruginosa* at different release times, respectively. As shown in Figure 8a, CEO’s inhibition zone towards *E. coli* reduced from 12.9 mm to 8.2 mm within release for 24 h, owing to the rapid evaporation of CEO. Furthermore, the antibacterial activity of CEO against *E. coli* was seriously diminished (the inhibition zone of only 7.8 mm) after storage for 30 d, owing to the volatilization of most CEOs. However, CEO-SNP still possessed strong antibacterial properties even after release for 30 d. The diameter of the inhibition zone slightly decreased from 13.6 mm to 11.3 mm. Figure 8c shows that the inhibition zone of CEO against *S. aureus* decreased from 20.7 to 15.2 mm at a release time of 24 h and only 9.4 mm was left after 30 d, owing to most of the CEO having been evaporated. Compared to CEO, the inhibition zone of CEO-SNP was only reduced by 3 mm within a release time of 24 h. Furthermore, CEO-SNP still retained obvious antibacterial effect after 30 d as there was still 15.9 mm left for the diameter of the inhibition zone. For Figure 8e,f, with the fast volatilization of CEO, the anti-bacterial effect of CEO against *P. aeruginosa* decreased obviously. After a release time of 30 d, the inhibition zone of CEO reduced from 12.4 to 9.0 mm. However, the antibacterial activity of CEO-SNP was not discounted and the inhibition zone of CEO-SNP only decreased from 13.1 mm to 13.0 mm after 30 d. The antibacterial activity results of SNP displayed that the blank SNP had no antibacterial activity against *E. coli*, *S. aureus* and *P. aeruginosa* (Figure 8g). These revealed that the volatility of CEO was significantly reduced, and the lasting antibacterial activity of CEO was improved after being adsorbed by the SNP carrier, which was consistent with the above analysis of the release and stability results.

#### 2.4.3. Antibacterial Mechanism Evaluation

**Figure 9:**
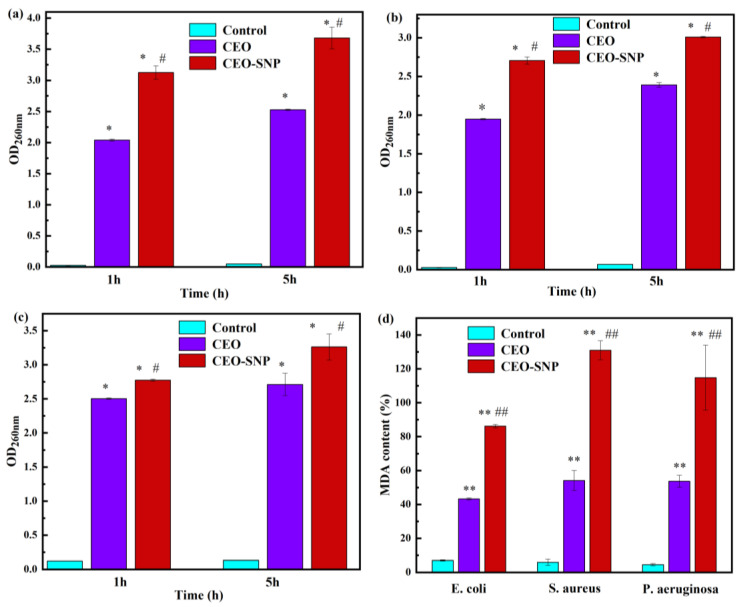
Nucleic acid contents of *E. coli* (**a**), *S. aureus* (**b**) and *P. aeruginosa* (**c**) suspensions after being treated by CEO and CEO-SNP, respectively. MDA content (**d**) of *E. coli*, *S. aureus* and *P. aeruginosa* after being treated by CEO and CEO-SNP, respectively. Values were expressed as mean ± S.D. (*n* = 3). * (*p* < 0.05) and ** (*p* < 0.01) represented the significant difference compared with control group; # (*p* < 0.05) and ## (*p* < 0.01) represented the significant difference between CEO-SNP and CEO group.

In order to clarify the antibacterial mechanism of CEO-SNP, the leakage of nucleic acid was studied via measuring the OD of the bacteria suspension at 260 nm. As an important constituent of bacteria, nucleic acid macromolecules were generally present in cells. However, once the bacterial structure was disrupted, the cellular contents leaked out and nucleic acid macromolecules were released. As shown in Figure 9a–c, after treatment of CEO-SNP and CEO for 1 h and 5 h, the absorbance values of the treated groups increased significantly compared with those of the control group, which meant that the cell membrane of the bacteria was damaged by CEO, causing the release of nucleic acid macromolecules and thus resulting in cell death [56,57]. As seen from Figure 9a–c, for all bacterial, the CEO-SNP treatment groups exhibited higher absorbance values than CEO treatment groups (*p* < 0.05). This indicated that the CEO-SNP might have a much stronger antibacterial effect compared with CEO.

**Figure 10:**
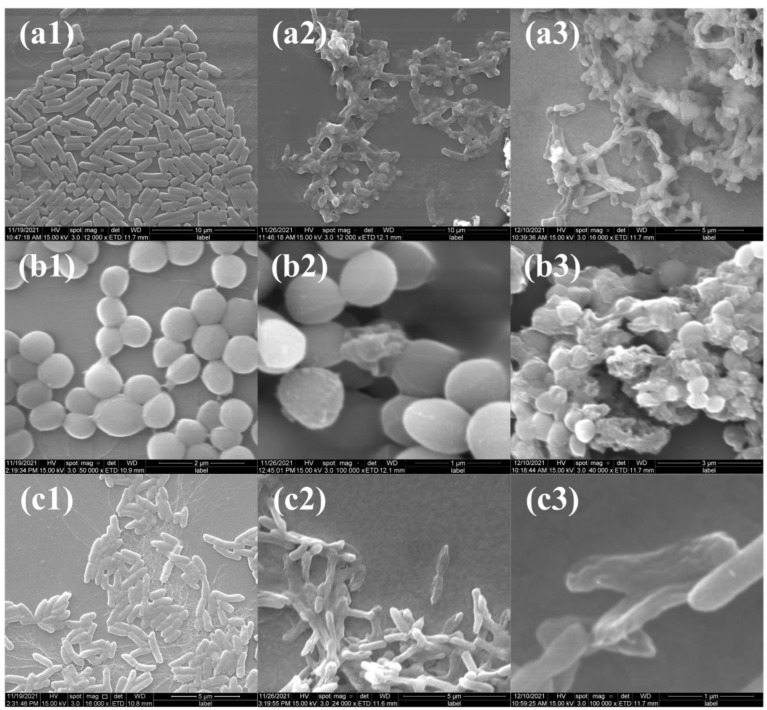
SEM images of *E. coli* (**a1**–**a3**), *S. aureus* (**b1**–**b3**) and *P. aeruginosa* (**c1**–**c3**). Control group (**a1**–**c1**), CEO groups (**a2**–**c2**), CEO-SNP groups (**a3**–**c3**).

The cell membrane disruption of bacteria was further confirmed by their morphology observation via SEM (Figure 10). Before antibacterial treatment, all bacteria exhibited a smooth surface, an intact cell membrane and a clear structure (Figure 10(a1–c1)). However, after treatment of CEO and CEO-SNP for 8 h, the morphology and membrane structure of the bacteria was strongly disrupted, and the cell surface obviously became rough, collapsed or even broken.

Malondialdehyde (MDA) is the end product of lipid peroxidation, and its contents are generally regarded as a parameter reflecting the degree of lipid peroxidation and injury to bacteria. The MDA content of *E. coli*, *S. aureus* and *P. aeruginosa* treated with CEO and CEO-SNP are shown in Figure 9d. In control group A, a significant increase in the amount of MDA was observed for the bacteria treated with CEO or CEO-SNP as compared to the control group, and the amount of MDA increased significantly (*p* < 0.01), suggesting that the membrane lipid of bacteria could be damaged via oxidative stress induced by CEO-SNP [58]. The result further elucidated the antibacterial mechanism of CEO-SNP that disrupted the cell structure and cell membranes. Moreover, a degree of membrane lipid peroxidation in *S. aureus* (gram-positive) was more serious than *E. coli* (gram-negative) and *P. aeruginosa* (gram-negative), which was consistent with the above results of the antimicrobial activity evaluation. Moreover, compared to that of the CEO treatment group, the MDA content of the CEO-SNP treatment group remarkably increased (*p* < 0.01), which meant the antimicrobial activity of CEO was enhanced, owing to the encapsulation of SNP.

**Figure 11:**
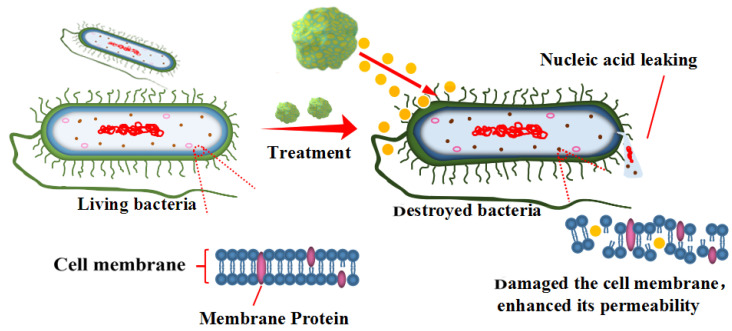
Schematic diagram of antibacterial mechanism of CEO-SNP.

Therefore, combined with the results of nucleic acid as well as MDA content determination and SEM images, the antibacterial mechanism of CEO-SNP was proposed as illustrated in Figure 11. After treatment with CEO-SNP, the oxidative stress was triggered and the ROS were excessively produced, which induced lipid peroxidation in the cytoplasmic membrane. Thereby, the cell membrane was damaged, leading to changes of the membrane permeability and leakage of cytoplasmic constituents, which ultimately leads to bacterial death [39,59,60].

## 3. Materials and Methods

### 3.1. Materials

*Chimonanthus nitens Oliv*. essential oil (CEO) was kindly donated by Jiangxi Youmei Pharmaceutical Co., Ltd. Pluronic 123 (P123), ammonium fluoride (NH4F), tetraethyl orthosilicate (TEOS, 98%), polyvinyl alcohol (PVA) and heptane (C7H16) were purchased from Sigma-Aldrich (Shanghai, China).

### 3.2. Synthesis of SNP

SNP were synthesized according to the method as described in the previous literature [61,62,63] with some modifications. P123 and PVA were used as a structure model agent. Firstly, P123 (24 g) and NH4F (0.27 g) were added into the 840 mL of 13 M HCl solution and mixed with PVA solution formed by dissolving 10 g PVA in 100 mL deionized water at 60 °C as template. Then, after adding 37 mL TEOS and 12 mL heptane, the template solution was stirred for 24 h at room temperature. The resulting solution was transferred to a closed-teflon container and reacted for another 24 h in a glycerin bath at 100 °C. After centrifugation, the synthesized SNP composites were separated, rinsed with deionized water and dried at 50 °C for 24 h. Finally, the SNP were harvested after calcination for 5 h at 550 °C.

### 3.3. Preparation of CEO-SNP

SNP (2 g) were mixed with 30 mL of CEO using an ultrasound (250 W, 40 kHz) for 5 min to disperse SNP uniformly and remove air bubbles in SNP pores. Then, the resulting mixture was further stirred at 800 rpm for 24 h at room temperature in order to facilitate the adsorption of CEO in the nanopores of SNP. Finally, the CEO-SNP was obtained after centrifugation and dried at room temperature.

### 3.4. Characterization of SNP and CEO-SNP

The structures of SNP and CEO-SNP were analyzed using FTIR Spectrometer (PerkinElmer, MA, USA) with a spectra wavelength range of 4000–500 cm^−1^. The Brunauer-Emmet-Teller (BET) adsorption-desorption isotherm was recorded with a Quadrasorb SI adsorption apparatus (APAP2460, Quantachrome, FL, USA) at 77 K. The samples were degassed at 473.15 K under a vacuum for about 12 h before analysis. The morphology was observed by means of scanning electron microscope (SEM) (SU8020, Hitachi, Tokyo, Japan) as well as transmission electron microscope (TEM) (FEI, OR, USA).

### 3.5. The Sustained-Release Characterization and Loading Capacity Evaluation of CEO-SNP

The sustained-release performance of CEO-SNP was investigated by recording weight loss rate and the remaining mass per second of a certain weight samples via a TGA thermogravimetric analyzer at a constant temperature of 25 °C and 40 °C for 12 h, respectively. The weight loss of CEO-SNP was measured using a thermogravimetric analyzer heating from 40 °C to 600 °C at a rate of 10 °C/min. The weight loss rate was equal to the loading capacity of CEO (LC) in the CEO-SNP. The cumulative release rate of CEO can be calculated by following equation:

R_t_ = (M − Mt)/(M × LC)

where R_t_ is the cumulative release rate of CEO; M_t_ is the remaining mass per second of samples; M is the initial mass of sample; and LC is the loading capacity of CEO.

### 3.6. Stability Test

CEO-SNP was stored in Stability Chambers (Labonce-720 CGS, Labonce, Beijing, China) with 40% relative humidity and adequate ventilation. The temperature was kept at 25 °C and 40 °C. Then, the 4.5–5.5 mg sample was taken at various time intervals and analyzed by thermogravimetry to record their weight loss and DTG curves for stability evaluation.

### 3.7. Anti-Bacterial Activity and Anti-Bacterial Mechanism Evaluation of CEO-SNP on E. coli, S. aureus and P. aeruginosa

#### 3.7.1. Culture of Bacterial Strains

The bacterial strains including *E. coli*, *P. aeruginosa* and *S. aureus* were purchased from BeNa Culture Collection China and stored at −80 °C in the Luria Bertani (LB) broth containing 25% glycerol. Three bacterial strains were placed in LB broth alone for 24 h at 37 °C. Colony McFarland turbidity of bacterial suspension after incubation was determined using Bacterial Turbidity Meter (WGZ-2XJ, Xinrui, Shanghai, China) and the colony forming unit (CFU) was adjusted to the desired cell density with a sterile PBS buffer solution.

#### 3.7.2. Assay of MBC of CEO

The bactericidal activity of the CEO was studied by the broth dilution method [64]. Briefly, the CEO was dissolved in 1% DMSO and diluted with LB broth to obtain serial dilutions (1.25 to 20 mg/mL CEO per tube). The bacterial suspension (1.0 × 10^7^ CFU/mL) was added into sterile test tubes and mixed with LB medium containing CEO at different concentrations. Then, the mixtures were cultured at 37 °C for 24 h. 1% DMSO was used as a negative control group. After incubation at 37 °C for 24 h, the minimum concentration that allows for no bacterial growth was considered as MBC. The determination was performed three times for each concentration.

The serial concentrations of CEO-SNP (1.25, 2.5, 5, 10, 20, 30 and 40 mg/mL) were obtained by dispersing in LB. Similarly, after inoculating with bacteria (1.0 × 10^7^ CFU/mL), the mixture was cultured for 24 h at 37 °C on a shaker bed at 150 rpm. Then, 100 μL of the mixture was inoculated onto nutrient agar (NA) plates and cultured at 37 °C for 24 h. The test tube containing SNP and nutrient broth was treated as a negative control group and each sample was performed in triplicate. The MBC was acquired through observing the plates.

#### 3.7.3. Long-Term Antibacterial Activity Evaluation of CEO-SNP

The long-term antibacterial performance of CEO-SNP was determined by the method described as previously reported [37] with slight modifications. Briefly, 100 μL of bacterial suspension (1.0 × 10^7^ CFU/mL) was evenly inoculated on LB agar plates (80 mm) with a diameter of 6 mm hole, and then the CEO-SNP containing 8.0 mg CEO was added into the hole in the center of the plate. The control group was treated by adding a 6 mm round scrap of paper that absorbed the same amount of CEO in the center of the LB plate. The LB plate was cultured for 24 h at 37 °C and the cross intersection method was used for measuring the diameter of the inhibition zone. The round scrap of paper containing CEO and the CEO-SNP was stored in the stability test chamber (25 °C, RH 40%). At predetermined time intervals, the samples were taken for the long-term antibacterial evaluation by determining the diameter of the inhibition zone.

#### 3.7.4. Nucleic Acid Detection of the Bacteria

In total, 4 mL *S. aureus*, *E. coli* and *P. aeruginosa* suspensions (1.0 × 10^7^ CFU/mL) were centrifuged and the bacteria were collected, rinsed three times with PBS buffer solution and resuspended in LB. Then, CEO-SNP was added, mixed and cultured at 37 °C for 1 h and 5 h; afterwards, the supernatant was instantly separated by centrifugation. A UV-VIS spectrophotometer (UV2550, SHIMADZU, Kyoto, Japan) was applied to determine the UV absorbance of the sample at 260 nm.

#### 3.7.5. MDA Content Detection of the Bacteria

The bacterial suspensions (1.0 × 10^7^ CFU/mL) were mixed with CEO-SNP, and cultured in shaker at 25 °C for 30 min at 150 rpm. The malondialdehyde (MDA) content was determined by commercial kits (Jiancheng, Naijing, China) according to the manufacturer’s instructions. The MDA content was measured by the colorimetric method as described in commercial kits.

#### 3.7.6. Morphology Evaluation of the Bacteria

The morphology of bacteria after treatment of CEO and CEO-SNP was evaluated by means of scanning electron microscope (SEM) based on the reported method [65]. The CEO-SNP was added into 1 mL bacterial suspension (10^7^ CFU/mL) and incubated in a shaker at 37 °C for 8 h. Then, the treated bacterial were fixed in 2.5% glutaraldehyde for 12 h at 4 °C and then washed three times with PBS buffer solution, followed by dehydration with gradient ethanol. After freeze-drying (SCIENTZ-10N, Ningbo, China), the morphology of the bacteria was evaluated by SEM.

## 4. Conclusions

In this study, novel sponge-liked silica nanoporous particles (SNP) loading CEO were successfully prepared using Pluronic 123 and PVA as a template and TEOS as a silica source. SNP had high thermal stability and a large pore volume and surface area, which offered ample space for more CEO loading. CEO-SNP could significantly delay the release of CEO, and the release behavior was in accordance with the first-order kinetic release model. CEO-SNP could markedly strengthen the antibacterial effect of CEO against *E. coli* and *S. aureus*, owing to destroying cell structure as well as cell membrane. Moreover, CEO-SNP exhibited a long-lasting antibacterial ability via controlling the sustained release of CEO. Therefore, the sponge-liked silica nanoporous particles seemed to be a promising carrier for CEO to acquire sustained-release and long-term antibacterial effects.

## Figures and Tables

**Table 1:** Pore structural parameters of SNP.

Analyzed Materials	Surface Area (m^2^/g)	Pore Volume (cm^3^/g)	Pore Diameter Dv (nm)
SNP	815.7278	1.804470	8.7026

**Table 2:** The Fitting Kinetic models for release behaviors of (a) CEO, (b) CEO-SNP.

T (°C)	Samples	Zero-Order Equation			First-Order Equation			Higuchi Equation			Korsmeyer–Peppas Equation		
		Y = k t + b			Y = k (1 − e ^b t^)			Y = k t ^1/2^ + b			Y = k t ^b^		
		k	b	R^2^	k	b	R^2^	k	b	R^2^	k	b	R^2^
25	CEO	1.0900 × 10^−3^	0.1899	0.8856	0.9327	−0.0034	0.9950	0.0376	−0.0889	0.9684	0.0230	0.5602	0.9600
	CEO-SNP	8.1820 × 10^−4^	0.1739	0.8749	0.7017	−0.0040	0.9990	0.0283	−0.0375	0.9678	0.0239	0.5163	0.9637
40	CEO	7.0912 × 10^−4^	0.5602	0.5215	0.9257	−0.0119	0.9930	0.0272	0.3298	0.7076	0.1921	0.2548	0.8086
	CEO-SNP	7.0348 × 10^−4^	0.435	0.6166	0.8055	−0.0097	0.9966	0.0262	0.2198	0.7931	0.1295	0.2939	0.8647

Where Y is the cumulative amount of drug released at time point, t represents time, X represents time, and k,b represent constant.

## Data Availability

Not applicable.

## Sample Availability

Samples of the compounds SNP, CEO and CEO-SNP are available from the authors.
